# Clinical utility of mesenchymal stem/stromal cells in regenerative medicine and cellular therapy

**DOI:** 10.1186/s13036-023-00361-9

**Published:** 2023-07-11

**Authors:** Vitali V. Maldonado, Neel H. Patel, Emma E. Smith, C. Lowry Barnes, Michael P. Gustafson, Raj R. Rao, Rebekah M. Samsonraj

**Affiliations:** 1 Department of Biomedical Engineering, University of Arkansas, 790 W Dickson St, Fayetteville, AR USA; 2 Department of Orthopedic Surgery, University of Arkansas for Medical Sciences, Little Rock, AR USA; 3 Nyberg Human Cellular Therapy Laboratory, Mayo Clinic, Phoenix, AZ USA; 4 Interdisciplinary Graduate Program in Cell and Molecular Biology, University of Arkansas, Fayetteville, AR USA

**Keywords:** Mesenchymal stem/stromal cells, MSCs, Clinical trials, Musculoskeletal, Cardiovascular, Nervous system, Immune system disorders, Regenerative medicine

## Abstract

Mesenchymal stem/stromal cells (MSCs) have been carefully examined to have tremendous potential in regenerative medicine. With their immunomodulatory and regenerative properties, MSCs have numerous applications within the clinical sector. MSCs have the properties of multilineage differentiation, paracrine signaling, and can be isolated from various tissues, which makes them a key candidate for applications in numerous organ systems. To accentuate the importance of MSC therapy for a range of clinical indications, this review highlights MSC-specific studies on the musculoskeletal, nervous, cardiovascular, and immune systems where most trials are reported. Furthermore, an updated list of the different types of MSCs used in clinical trials, as well as the key characteristics of each type of MSCs are included. Many of the studies mentioned revolve around the properties of MSC, such as exosome usage and MSC co-cultures with other cell types. It is worth noting that MSC clinical usage is not limited to these four systems, and MSCs continue to be tested to repair, regenerate, or modulate other diseased or injured organ systems. This review provides an updated compilation of MSCs in clinical trials that paves the way for improvement in the field of MSC therapy.

## Background

Mesenchymal stem/stromal cells (MSCs) are somatic stem cells that have the capacity for self-renewal, multilineage differentiation, and immunomodulation. MSCs can be isolated from various sources, including bone marrow, adipose tissue, umbilical cord, cord blood, placenta, among other tissue sources. Originally MSCs were identified as stromal or support cells for the hematopoietic stem cells in the bone marrow. The ease of isolation and their accessibility make MSCs a desirable source for different clinical applications. Numerous studies showcase the immunomodulatory and homeostatic roles of MSCs in inflammation regulation, exhibiting this immunomodulatory regulation through cell–cell contact and paracrine signaling [1, 2]. MSCs also exhibit growth factor secretion and can traffic towards injured areas [3]. These properties, combined with the ease of in vitro expansion, make MSCs suitable candidates for experimental research, pre-clinical studies and clinical trials. The current review briefly discusses the key characteristics of MSCs from various tissue sources, and summarizes key evidence of therapeutic potential for musculoskeletal, nervous, cardiovascular, and immune repair, highlighting the clinical utility of MSCs in treating some common disorders within each organ system considered.

### Identity, characterization and tissue sources of MSCs

The International Society of Cellular Therapy (ISCT) put four minimum criteria for defining mesenchymal stem/stromal cells [4], which include fibroblast-like morphology, plastic adherence, multilineage and multipotential capacity for differentiation into osteoblasts, adipocytes, and chondrocytes, along with expression of cell surface proteins CD73, CD90 and CD105, and lacking expression of lineage-specific markers CD45, CD34, CD14, CD19, CD11b, and HLA-DR. In earlier investigations, MSCs were predominantly obtained from bone marrow; however, as research on MSCs expanded, other tissue sources were identified, which include adipose tissue, placenta, amniotic fluid, umbilical cord, dental pulp, to name a few. Furthermore, these different sources give rise to MSCs with unique characteristics.

MSCs derived from bone marrow (BM–MSCs) are considered the most widely used and exhibit all the typical characteristics of MSCs [5, 6]. Cells from this source have been used in more clinical trials than MSCs derived from any other source based on data available from clinicaltrials.gov. However, they cannot be easily obtained since the donor must undergo a painful and invasive procedure of bone marrow aspiration usually from the iliac crest. This motivated researchers to search for alternative sources of MSCs that could be more easily obtained in larger quantities. Adipose tissue has emerged as one of the major alternative sources of allogeneic MSCs and are being extensively investigated owing to their ease of isolation and availability in large tissue quantities useful for procurement of sufficient primary cells. Adipose MSCs (Ad-MSCs) can differentiate into various types of cells like adipocytes, osteoblasts, myocytes, chondrocytes, neural cells, hepatocytes, epithelial cells, and endothelial cells [7]. Characterization of Ad-MSCs revealed identification of non-classical markers including CD36, CD200, and CD274 [8]. Ad-MSCs require greater doses of growth factors (TGF-β and IGF-1) to have a comparable chondrogenic differentiation to the one observed in BM-MSCs [9].

MSCs derived from the placenta are readily sourced and available in abundance. In addition to expressing the classic cell surface markers satisfying the ISCT minimal criteria, placental MSCs (P-MSCs) are positive for UEA-1 (which is negative in BM-MSCs), CD166, CD73, CD44, CD105, CD29, and HLA-ABC while being negative for CD31, CD34, CD14, CD45, and HLA-DR. In addition, these cells also express renin and flt-1, which are not expressed in BM-MSCs [10]. Placental MSCs can be differentiated into multiple cell types successfully. However, the proliferative capacities of MSCs derived from the same placenta are heterogenous, with some placental cells showing a proliferative capacity of more than twenty passages, while others only proliferate between ten and twenty passages [11]. Another suitable alternative source of MSCs is human amniotic fluid (AF-MSCs). Moreover, MSCs derived from this source are better at self-renewing and have a higher and faster proliferative capacity than BM-MSCs. Additionally, they have a higher capacity to differentiate into hepatic cells and express liver-specific markers. In addition, they have the same gene stability and immunophenotype as that of BM-MSCs [12].

Umbilical cord and cord blood is a convenient source of MSCs because it can be easily harvested, and there is no significant difference between umbilical cord blood-derived MSCs (UCB-MSCs) and BM-MSCs in terms of immunophenotype and morphology. However, UCB-MSCs have a lower expression of CD105 and CD90; and a lower colony frequency compared to BM-MSCs. Also, UCB-MSCs have no adipogenic differentiation capacity. Nevertheless, UCB-MSCs can be cultured for a longer time and have a higher capacity to proliferate compared to BM-MSCs [13]. A disadvantage of UCB-derived MSCs is that the isolation efficiency is low. On the contrary, umbilical cord-derived MSCs (UC-MSCs) are much more efficient at being isolated [14]. UC-MSCs are similar to BM-MSCs in gene expression: when comparing abundant transcripts between the two cell types, only 0.8% of tags found in UC-MSCs are not found in BM-MSCs. On the other hand, only 2.9% of tags found in BM-MSCs are not found in UC-MSCs [15].

Dental pulp can be effective source for stem cells since stem cells from deciduous teeth pulp have a higher proliferative capacity than BM-MSCs. Also, stem cells derived from dental pulp (DP-SCs) have a higher expression of the basic fibroblast growth factor, BMP-2, RUNX2, and ALP genes. In contrast, BM-MSCs have a higher osteogenic differentiation capacity and higher expression of alkaline phosphatase [16]. DP-SCs show more odontogenic differentiation capacity than bone marrow stromal stem cells and have greater mineralization rates. Moreover, DP-SCs express multiple stem cell crest-derived surface markers, like GFAP, HNK-1, nestin, P75, and S-100, suggesting that they are derived from cranial neural crest cells [17].

### Therapeutic applications of mesenchymal stem cells

**Fig. 1:**
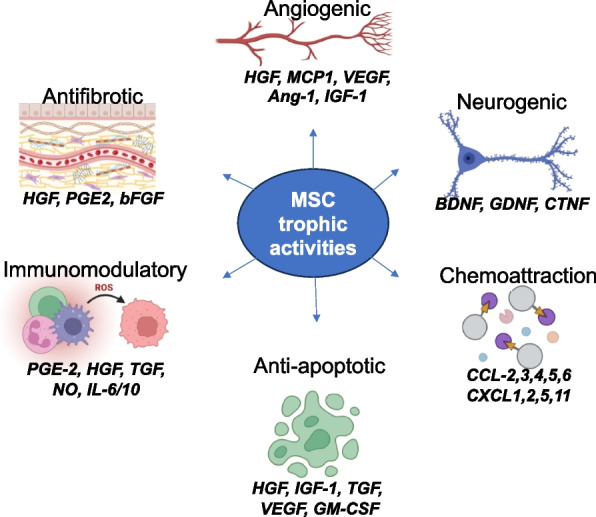
Functionality of MSCs: Schematic of key trophic functions of mesenchymal stem cells and participating bioactive factors in tissue repair and immunomodulation

**Fig. 2:**
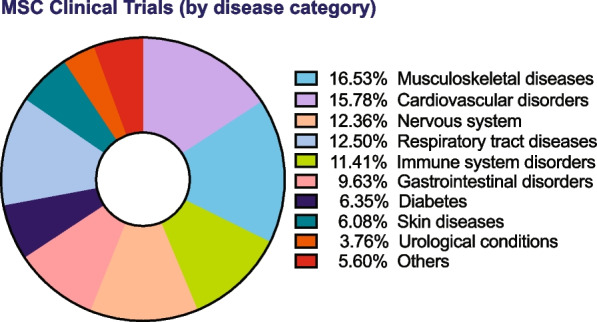
MSC clinical trials by disease category: Schematic representation of recorded clinical trials utilizing mesenchymal stem cells across multiple organ systems and related disorders. Pie chart was plotted using MSC clinical trial numbers obtained from clinical trials.gov (as of March 2023) and computing percentage distribution of MSC trials across various disease categories

**Fig. 3:**
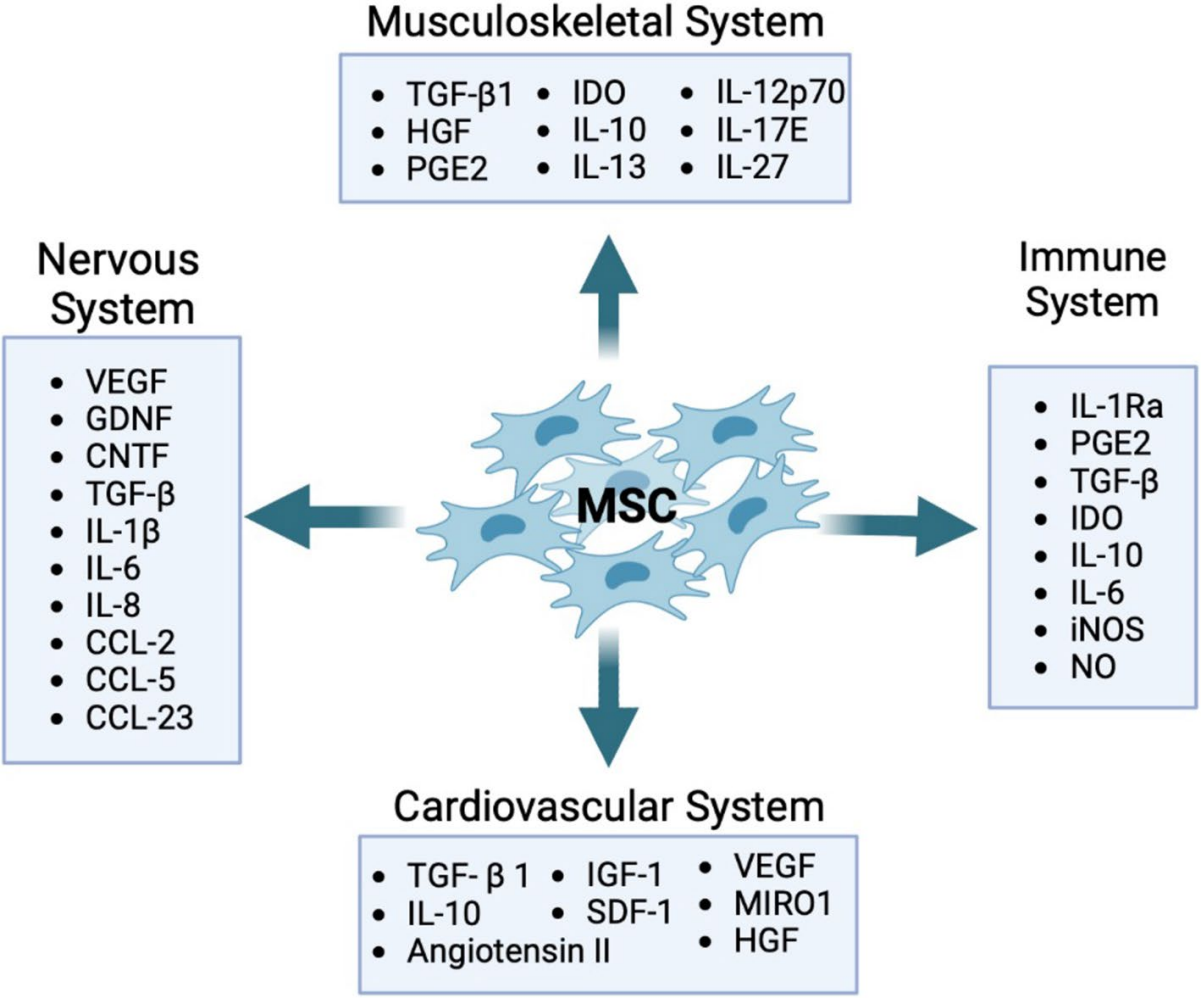
Key disease systems managed by MSCs in clinical applications: Schematic showing significant molecular players involved in repair and regeneration of tissues of the musculoskeletal, nervous, immune, and cardiovascular systems

Recent research has provided evidence that MSCs exert therapeutic effects not only by engraftment and differentiation but also through the secretion of biologically active molecules that exert beneficial effects on other cells. MSC paracrine effects can be broadly classified into trophic, immunomodulatory, and chemoattraction. Specifically, secreted factors from MSCs are known to mediate angiogenic, mitogenic, anti-fibrotic, anti-apoptotic, anti-scarring, and neurogenic functions (Fig. 1). Researchers have been actively using MSCs in numerous studies, with more than 1476 clinical trials listed in clinicaltrials.gov (as of March 2023) of which a majority are targeted at treating disorders of the musculoskeletal, nervous, cardiovascular, and immune-related disorders (Fig. 2). The key molecular players involved in regeneration or repair of each of the disease systems are represented schematically (Fig. 3) In this review, we will discuss outcomes of preclinical and clinical studies utilizing MSCs for these four major disease categories. The sources of MSCs used for specific clinical applications are tabulated in Table 1.

**Table 1:** List of key studies utilizing MSCs for treating various clinical conditions

**Musculoskeletal Disease**				
	Study Number	Type of MSC used in the study	Main Effect	References
	1	UC-MSCs	Stable muscle power after 1-year follow-up. No negative effects	[17]
	2	BM-MSCs	Improve tissue repair and protect muscles from damage induced injury	[18]
	3	UC-MSCs	IGF-1 and MSCs injection led to efficient repair of muscles in mouse model	[19]
Muscular Dystrophy	4	UC-MSCs	Myogenic differentiation of MSCs combine with ray myoblasts to form myotubules	[20]
	5	P-MSCs	Mice showed increased levels of utrophin and reduced inflammation	[21]
	6	BM-MSCs	Dystrophin expression chimeric MSCs increased function and strength of muscle and less immune response	[22]
	1	iPSC-derived MSCs	Bone loss reduction and micro vessel density in mouse model	[23]
	2	BM-MSCs	Reduced total hip replacement arthroplasty conversion rate	[24]
Osteonecrosis	3	BM-MSCs	Suppressed adipogenesis and upregulated SOX9	[25]
	4	BM-MSCs	Facilitated repair of bone	[26]
	5	Rabbit MSCs	MSCs with GFP tag, fluorescence only on the femur	[27]
	1	BM-MSCs	Higher bone mineral density and accelerated cranial bone healing	[28]
	2	Ad-MSCs and UC-MSCs	Adipose MSCs exhibit higher osteogenic differentiation and higher bone formation rates	[29]
Cranial Defects	3	UC-MSCs and BM-MSCs	Rats treated with MSC treatment exhibit greater expression of Runx2, collagen I, and osteocalcin	[30]
	4	DP-MSCs and BM-MSCs	Dental pulp MSCs exhibit similar properties as BM-MSCs for bone regenerative applications	[31]
	5	Ecto-MSCs and BM-MSCs	Ecto-MSCs had a higher number of proliferative cells, but both cells promoted bone regeneration	[32]
	1	Ad-MSCs	Osteogenic differentiation and vascularization in non-union fracture models in rats	[33]
	2	UC-MSCs	Optimal bone formation in infected non-union fracture in 54 year old patient	[34]
Non Union Bone Fracture	3	BM-MSCs	BM-MSCs into non-union femur fracture showed bone union in 8 weeks	[35]
	4	BM-MSCs	MSCs combined with shock-wave therapy improved fracture stiffness and mechanical strength	[36]
	5	BM-MSCs	Exosomes from BM-MSCs enhanced osteogenesis, bone healing, and angiogenesis	[37]
	1	BM-MSCs	Hyaluronic acid and BM-MSCs showed MOCART scores than patients with only hyaluronic acid treatment	[38]
Osteoarthritis	2	Ad-MSCs	Improved pain and knee function of patients with no negative effects	[39]
	3	Ad-MSCs	Injection of 10^8 MSCs found effective to reduce osteoarthritis symptoms	[40]
**Nervous System Diseases**				
	Study Number	Type of MSC used in study	Main Effect	References
	1	UCB-MSC	Activation of microglial cells and prevention of β-amyloid peptide plaques deposition. Induce endogenous neurogenesis	[41]
	2	BM-MSC	Ameliorate Alzheimer’s disease through induction of miR-29c-3p and targeting of BACE1	[42]
Alzheimer’s Disease	3	Unknown source MSC	Promote neurogenesis and relieve Aβ 1–42 induced cognitive impairment	[43]
	4	BM-MSC	Reduce astrocytic inflammation and synaptogenesis. Increase expression of microRNA-146a in hippocampus	[44]
	5	MB-MSC	Memory and spatial learning improvement. Reduction of tau hyperphosphorylation. Improving of amyloid plaques. Increase in Aβ degrading enzymes and reduction of pro-inflammatory cytokines	[45]
	1	UC-MSC	Ameliorate neuroinflammation and locomotor functions. Preservation of intestinal goblet cells	[46]
	2	BM-MSC	Reduction in expression of hydroxylase immunoreactive cells and neural loss	[47]
Parkinson’s Disease	3	WJ-MSC	Restoration of BDNF, NGF, passive avoidance, memory, and hippocampal long-term potentiation	[48]
	4	UC-MSC	Reduction in cell apoptosis and loss of dopaminergic neurons	[49]
	5	BM-MSC	Increase in the number of TH-positive neuronal cells and fibers	[50]
	1	BM-MSC	Slower increment in Unified MSA Rating Scale score	[51]
	2	UCB-MSC	Lower Unified MSA Rating Scale score than the control group	[52]
Multiple System Atrophy	3	BM-MSC	Prevention of neurodegeneration and improvement in behavioral disorders	[53]
	4	Ad-MSC	Lower Unified MSA Rating Scale score than control group	[54]
	5	BM-MSC	Increase survival rate of nigrostriatal neurons	[55]
	1	WJ-MSC	Showed no adverse effects	[56]
	2	BM-MSC or Ad-MSC	Lowered ALS Functional Rating Scale-Revived score and the forced vital capacity	[57]
Amyotrophic Lateral Sclerosis	3	WJ-MSC	Increase in survival time	[58]
	4	BM-MSC	Reduction of ALS Functional Rating Scale. Increase in anti-inflammatory and decrease in pro-inflammatory cytokines	[59]
	5	BM-MSC	Improvement in neurodegeneration, neuroinflammation, and neurotrophic factors	[60]
	1	UC-MSC	Improved sensory functions and mobility	[61]
	2	UC-MSC	Improved motor and sensory functions	[62]
Spinal Cord Injury	3	Ad-MSC	Improved motor and sensory functions	[63]
	4	BM-MSC	Showed no adverse effects	[64]
	5	UC-MSC	SCI recovery and nerve growth factor production	[65]
**Cardiovascular Diseases**				
	Study Number	Type of MSC used in the study	Main Effect	References
	1	Unknown source MSCs	Extracellular vesicles restored right ventricular systolic pressure to baseline	[66]
	2	iPS-MSCs	Extracellular vesicles reduced arterial stiffness and hypertension in mice models. Also, promoted the expression of AMPKα, eNOS, and SIRT1	[67]
Hypertension	3	BM-MSCs	MSC transplantation improved collagen deposition and attenuated EndMT and factor-2α	[68]
	4	Skin-Derived MSCs	Decreased vascular damage and systolic blood pressure. Reduced Th17 cells in peripheral blood in a mouse model	[69]
	5	UC-MSCs	Exosomes lessened hypertrophy in the right ventricle and caused pulmonary vascular remodeling in a rat model	[70]
	1	Ad-MSCs	Exosomes suppressed cardiac dysfunction and apoptosis, and increases in M2 polarization was observed	[71]
	2	BM-MSCs	Improved angiogenesis and cell survival in mouse model	[72]
Myocardial Infarction	3	Unknown source MSCs	Exosomes from SDF1 overexpressing MSCs increased microvascular restoration in endothelial cells and inhibited apoptosis of myocardial cells	[73]
	4	BM-MSCs	Anti-mR-155-5p MScs improved angiogenesis and cell survival even compared to control MSCs and no MSCs in the mouse model	[72]
	5	UC-MSCs	Exosomes from TIMP2 overexpressed MSCs promoted angiogenesis at the infarction site in a rat model	[74]
	1	BM-MSCs	Injection of exosomes from MSCs improved neurogenesis and angiogenesis in mouse models. Reduction of IL-1β expression was also observed	[75]
	2	UC-MSCs	Exosomes reduced inflammation in vitro and lowered infarct volume. Enhanced activation of microglia	[76]
Stroke	3	Various sources MSCs	MSCs as adjuvant therapy improved motor functions in to lower extremities in middle cerebral artery infarction sites	[77]
	4	BM-MSCs	MSCs’ extracellular vesicles promoted angiogenesis and neurogenesis in the stroke model	[78]
	5	BM-MSCs	Exosomes improved neurological function and enhanced neuroprotective effects in type 2 diabetic rat stroke model	[79]
	1	UC-MSCs	Administration of MSCs increased expression of hepatocyte growth factor	[80]
	2	Unknown source MSCs	MSCs overexpressing adrenomedullin enhanced heart function and increased cell survival in rat model	[81]
Heart Failure	3	Human Amniotic MSCs	Nanoparticle labeled MSCs increased cell homing and enhanced myocardial hypertrophy and heart function in a rat model	[82]
	4	Ad-MSCs	Enhancing AD-MSC exosomes through adiponectin treatment improved cardiac function and reduced inflammation and fibrosis in a mouse model	[83]
	5	BM-MSCs	Injection of BM-MSCs reduced myocardial infarction size and interstitial fibrosis thus enhancing heart rate variability	[84]
	1	UC-MSCs	MSCs lowered the percentage of infarct size change	[85]
	2	BM-MSCs and UC-MSCs	Transplantation of MSCs along with Coronary Arter Bypass Grafting Surgery showed decline in NT-proBNP	[86]
Chronic Ischemic Cardiomyopathy	3	BM-MSCs	MSC infusion significantly increases and improves cardiac function	[87]
	4	BM-MSCs and UC-MSCs	Intramyocardial injection of MSCs improved left ventricle function in 1, 3, 6 and 12 month followups	[88]
	5	BM-MSCs	Intramyocardial injection of MSCs significantly improved regional function of left ventricle in 3 and 6 months	[89]
**Immune-related diseases**				
	Study Number	Type of MSC used in study	Main Effect	References
	1	BM-MSC	Decreased blood glucose. Increased C-peptide and insulin	[90]
	2	UC-MSC	TGFBI is crucial for MSCs to suppress T-cell proliferation	[91]
Type 1 Diabetes Mellitus	3	BM-MSC	Increase in Langerhans islets diameter and amount of zymogen granules	[92]
	4	Ad-MSCs	Maintenance of viability and insulin secretion of pancreatis islets	[93]
	5	UC-MSC	Tissue repair of damaged islets. Decrease in blood glucose levels and lesions in renal tissue	[94]
	1	UC-MSC	Decrease in blood globulin and platelet level. Decrease in erythrocyte sedimentation rate, C-reactive protein level, and rheumatoid factor	[95]
	2	UC-MSC	Decrease in Desease Activity Score-28. Decrease in IL-6 and increase in IL-10. A higher Treg to Th17 ratio	[96]
Rheumatoid Arthritis	3	UCB-MSC	Decrease in IL-8, IL-6, IL-1β, and TNF-α	[97]
	4	BM-MSC	Decrease microRNA-584e. Reduction in NF-κB activity	[98]
	5	BM-MSC	Greater improvements in knee injury	[99]
	1	UC-MSC	Decrease in SLE Disease Activity Index. Improvements in levels of serum albumin	[100]
	2	BM-MSC	Increase in CD4 + Foxp3 + regulatory T-cell percentage. Improvement in Glomerular filtration rate, SLE Disease Activity Index, and serological tests	[101]
Systematic Lupus Erythematosus	3	UC-MSC	Increase in regulatory T-cells. Balance Th1 and Th2 cytokines	[102]
	4	UC-MSC	Increase in IFN-γ levels and a decrease in IL-6 levels	[103]
	5	UC-MSC	Downregulate inflammatory genes while upregulating miR-181a	[104]
	1	BM-MSC	Remission of GVHD	[105]
	2	BM-MSC	Decrease in number of Th1 cells	[106]
Graft vs. Host Disease	3	UC-MSC	Cured cGVHD. Increase in CD4 + CD25 + CD127 − regulatory T cells. Decrease in non-killer cells	[107]
	4	BM-MSC	Increase in overall survival time	[108]
	5	BM-MSC	Increase in regulatory T-cells and B cells	[109]

### Musculoskeletal applications

Multiple conditions affecting the musculoskeletal system can have life-long effects on the patients. To find a potential treatment for these conditions, researchers have explored and demonstrated the regenerative abilities of MSCs. Because of their efficacy in regenerating and repairing bone, tendons, joints, and skeletal muscles, several trials have been performed. Common disorders/injuries undergoing clinical trials with MSCs include muscular dystrophy, osteonecrosis, cranial defects, non-union bone fractures, and osteoarthritis.

#### Muscular dystrophy

Muscular dystrophy (MD) is a common neuromuscular disorder and is characterized by progressive degeneration of muscle resulting in muscular weakness [18]. For every 10,000 males between the ages of 5 and 24, 1.38 have Duchenne or Becker MD in the USA [19].

The transplantation of MSCs could be a possible treatment for MD since many clinical trials with these cells showed positive outcomes. The transplantation of human UC-MSCs in patients with Duchenne’s Muscular Dystrophy (DMD) resulted in stable muscle power in one-year follow-up, without any negative effects, like graft-versus-host disease [20]. In an animal study, IL-10 expressing AAV vector-transduced rat BM-MSCs (IL-10 MSCs) were shown to maintain long-term engraftment and help with tissue repair in mice. Furthermore, IL-10 MSCs protected muscles from damage-induced injury thereby improving muscle malfunction in DMD [21]. Likewise, combined IGF-1 and human UC-MSCs injection into a dystrophic mouse model promoted efficient repair of the muscles, thus, improving muscle strength. The combination also reduced fibrosis and inflammation of muscles [22]. The combination of MyoD (regulator of differentiation into the skeletal muscle) and human UC-MSCs showed myogenic differentiation of MSCs as early as five days after treatment with MyoD. Nevertheless, these cells were also able to combine with primary rat myoblasts and form heterokaryotic myotubules [110]. Similarly, P-MSCs and their exosomes lowered levels of creatine kinase, fibrosis, and expression of TGF-β on the cardiac muscles and diaphragm of an mdx mouse model of Duchenne MD. Also, the mice showed increased levels of utrophin and a reduction in inflammation [111]. Finally, treatment with dystrophin expressing chimeric (DEC) BM-MSCs myoblasts on an mdx mouse model of Duchenne MD increased the function and strength of the muscle and lowered immune response [112].

#### Osteonecrosis

Osteonecrosis, also known as aseptic necrosis, avascular necrosis, or ischemic bone necrosis, is one of the common bone degenerative diseases in the U.S., with about 10,000–20,000 new cases every year [23]. In osteonecrosis, the deficiency of blood flow to the bone is followed by cell death at the site, resulting in severe pain [24]. Although the most common type of osteonecrosis is the osteonecrosis of the femoral head, it can happen in other bones like the bones of the shoulders, ankle, and knee [25].

Surgical and non-surgical interventions are available for the treatment of osteonecrosis. However, researchers are searching for better alternatives, and MSCs have continued to remain as promising candidates for treatment of musculoskeletal disorders. Intravenous injection of induced pluripotent stem cells (iPSC)-derived MSCs showed a significant reduction of bone loss and an increase in microvessel density at the femoral head in a mouse model with steroid-induced osteonecrosis which was attributed to the induction of angiogenesis [26]. Likewise, BM-MSCs implantation on a femoral head with early-stage osteonecrosis reduced the total hip replacement arthroplasty conversion rate [27]. Similarly, exosomes isolated from BM-MSCs harvested from healthy rats when incubated with MSCs from rats with steroid-induced necrosis of the femoral head suppressed adipogenesis and upregulated SOX9 in the later rats. This might be because MSC’s exosomes induce osteogenesis in patients with osteonecrosis [113]. Next, a composite implant containing BM-MSCs, carboxymethyl chitosan, endothelial progenitor cells, and alginate facilitated the repair of the bone through angiogenesis and osteogenesis induction and reduced adipogenesis on steroid-induced osteonecrosis of the femoral head rabbit model [28]. Finally, intravenous administration of methylprednisolone stimulated rabbit-derived MSCs labeled with a green fluorescent protein showed the expression of the green fluorescent protein only on the femur, suggesting that MSCs can be utilized in preventing osteonecrosis [29].

#### Cranial defects

Cranial and craniofacial defects are conditions characterized by the inappropriate migration, formation, and differentiation of the neural crest-derived cells, causing malformed, small, or missing craniofacial bones. These conditions are common, with approximately one-third of all birth defects consisting of craniofacial abnormalities [30].

The effectiveness of MSC treatment for cranial defects is under investigation, with numerous successful outcomes in animal studies. The rats with cranial defects, when treated with BM-MSCs, had a greater bone mineral density, higher expression of osteocytes and osteoclasts, and accelerated cranial bone healing [31]. A comparative study on the osteogenic differentiation capacity between Ad-MSCs and human UC-MSCs in vitro and in vivo using rats with cranial defects showed that Ad-MSCs have higher osteogenic differentiation and a higher rate of bone formation in the cranial bone of the treated rats [32]. Likewise, the rats with cranial defects seeded with human UC-MSCs and human BM-MSCs had a greater expression of Runx2, collagen I, alkaline phosphate, and osteocalcin. They also had higher quantities of new bone and blood vessels than the control group [114]. More recent comparison studies, between dental pulp MSCs (DP-MSCs) and BM-MSCs, have shown that DP-MSCs implanted in rabbit calvarial defects model exhibit similar bone regeneration efficacy as BM-MSCs. Furthermore, DP-MSCs are easier to collect and more accessible than BM-MSCs. The results showed that in-vivo treatment of DP-MSCs had similar bone mineral density, new bone formation, and osteogenic protein expression [115]. Finally, the comparison of the therapeutic potential of ecto-MSCs (MSCs derived from human embryonic stem cells) and BM-MSCs in a calvarial defect rat model proved that the ecto-MSC treated group had higher cellularity and a higher number of proliferative cells. However, both cells (implanted on a scaffold) promoted regeneration of the bone [33].

#### Non-union bone fractures

The American Food and Drug Administration (FDA) defines a non-union bone fracture as any fracture that persists for at least nine months without signs of healing for three months. Non-union bone fractures comprise nearly 4.9% of all bone fractures [34]. Nevertheless, these fractures do not heal without medical intervention [35]. This highlights the need for a regenerative approach for non-union bone fracture treatment.

MSC therapy for non-union bone fractures have been trialed extensively with notable successes. Combination of Ad-MSCs, Chitosan hydrogen, and cancellous bone graft showed Vegf and Bmp2 gene expression, as well as osteogenic differentiation and vascularization in non-union fracture models in rats. This indicates that Ad-MSCs have positive effects on bone reconstruction and induction of bone cells at the injury site [36]. Similarly, the combination of UC-MSCs, BMP-2, and Hydroxyapatite in an infected non-union fracture of a 54-year-old patient showed faster and more optimal bone formation at the disease site with no side effects [37]. Also, transplantation of cell sheets of rat BM-MSCs into a non-union femur fracture rat model showed bone union in eight weeks [116]. Likewise, BM-MSCs, when combined with extracorporeal shock-wave therapy in a rabbit model of nonunion bone fracture, improved fracture stiffness, mechanical strength, and histological scores [117]. Finally, injection of exosomes derived from BM-MSCs into the site of the nonunion femoral bone fracture rat model once every week enhanced osteogenesis, bone healing processes, and angiogenesis [38].

#### Osteoarthritis

Osteoarthritis (OA), a bone degenerative disease, affects more than 32 million adults in the U.S [39]. The characteristics of this disease include articular cartilage degeneration, changes in subchondral and peri-articular bone, and limited intraarticular inflammation accompanied by synovitis [40].

Many studies have analyzed the effects of MSCs in the treatment of OA, and multiple clinical trials have shown positive outcomes. The intra-articular injection of hyaluronic acid combined with cultured BM-MSCs into the patients with osteoarthritic knee after surgery showed a better magnetic resonance observation of cartilage repair tissue (MOCART) scores in comparison to the patients receiving only hyaluronic acid [118]. Additionally, intra-articular injection of Ad-MSCs into patients with OA in the knee improved the pain and knee function of patients and had no negative effects. This improvement in pain and knee function can be attributed to a decrease in cartilage defects and an increase in the cartilage volume at the site [119]. Moreover, an injection dose of 10^8^ MSCs was found to be the most effective in reducing OA symptoms [120].

### Nervous system applications

Many of the diseases affecting nervous systems are neurodegenerative in nature. Neurodegenerative diseases occur due to the progressive loss of function and ultimate death of neurons. Though symptomatic cures for these diseases are available, no known cures are available to date. Thus, scientists are researching regenerative approaches to treat neurodegenerative disorders wherein MSCs have been trialed as suitable candidates owing to their ability to be differentiate into neurons in vitro. However, their in vivo effectiveness and potency for nerve repair are still being investigated in preclinical and clinical studies. Some clinical trials have shown that MSC treatment improves survival rates, reduces pathology, rescues the decline of cognitive functions, ameliorates disease symptoms, and reduces relapse occurrences [121]. Common neurodegenerative diseases undergoing clinical trials include Alzheimer’s disease, amyotrophic lateral sclerosis (ALS), multiple system atrophy (MSA), Parkinson’s disease (PD), and spinal cord injury (SCI).

#### Alzheimer’s disease

Alzheimer’s disease causes dementia, which is characterized by a decline in language, memory, cognitive skills, and problem-solving abilities of the affected patient. This disrupts the person’s lifestyle and is fatal over time. The disorder is caused by the damage or destruction of nerve cells in specific parts of the brain [122]. In the U.S., around 121,000 deaths caused by Alzheimer’s disease were recorded in 2019 [123], and as many as 6.2 million may have Alzheimer’s disease according to a report from the Alzheimer’s Disease Association in 2022 [41].

Considering regenerative medicine approaches to treat Alzheimer’s disease, MSCs have been proposed as promising cellular therapy candidates owing to their observed ability to halt disease progression and regenerate damaged neural tissue [42, 43]. There is evidence that UCB-MSCs prevented deposition of the β-amyloid peptide (Aβ) plaques and activated microglial cells. MSCs were also noted to induce endogenous neurogenesis, which can preserve or restore the cognitive functions of patients with Alzheimer’s disease [44]. Similarly, BM-MSC derived EV can ameliorate Alzheimer’s disease through targeting BACE1 through miR-29c-3p introduction to the neurons. This helps in activating the Wnt/β-catenin pathway [45]. Additional evidence that transplantation of MSC-derived exosomes relieved Aβ 1–42 induced cognitive impairment and promoted neurogenesis in a mouse model with Alzheimer’s disease supports the regenerative potential of MSC-derived products, like exosomes [124]. Additionally, intracerebroventricular injection of BM-MSCs into an Alzheimer’s mouse model ameliorated cognitive impairment by reducing synaptogenesis and astrocytic inflammation. The treated mice showed increased microRNA-146a expression in the hippocampus, which is most likely involved in the improvement of cognitive impairment [46]. Finally, transplantation of menstrual blood-derived MSCs (MB-MSCs) intracerebrally into a mouse model improved memory and spatial learning, reduced tau hyperphosphorylation, and improved amyloid plaques, while increasing Aβ degrading enzymes and reduced pro-inflammatory cytokines [47]. Together, these studies support the clinical potential and utility of MSCs in treating neurodegenerative disorders.

#### Parkinson’s disease

PD is a neurodegenerative disease characterized by the loss of dopaminergic neurons in the substantia nigra. In 2019, there were around 35,000 deaths attributed to PD in the U.S. alone [123]. Currently, there is no neuroprotective treatment or cure available for treating PD [48].

Reports on cellular therapy using MSCs for treating PD have yielded several promising outcomes. The use of UC-MSCs in PD rodent models ameliorated locomotor deficit as well as neuroinflammation. This allowed the mouse to preserve more dopaminergic neurons. Moreover, the administered MSCs allowed the mouse to preserve intestinal goblet cells which protected the host from pathogens [49]. Similarly, human BM-MSCs reduced neural loss and the expression of hydroxylase immunoreactive cells in vivo. This study suggested that human MSCs have a neuroprotective effect and prevent the loss of dopaminergic neurons [50]. Moreover, administration of Wharton’s Jelly-derived MSCs (WJ-MSCs) into PD rat models caused restoration of BDNF and NGF, as well as hippocampal long-term potentiation, passive avoidance, and memory [125]. In another rat model, delivery of human UC-MSC-derived exosomes resulted in successful biodistribution to the substantia nigra of the brain, resulting in the reduction of the loss of dopaminergic neurons and apoptosis. Additionally, increased dopamine levels in the striatum were observed accompanied by relieving apomorphine-induced asymmetric rotation [126]. Further developments in research document the post-treatment effects of administering rat BM-MSCs to the substantia nigra in a rat model wherein double immunofluorescence and immunohistochemical assessments revealed increases in the number of TH positive neuronal cells and fibers, as well as functionality, compared to non-treated controls [51].

#### Multiple system atrophy

MSA is a fatal neurodegenerative disease characterized by parkinsonian elements, autonomic malfunction, cerebellar features, and pyramidal features [52]. The prevalence of Multiple System Atrophy is around 4.4 per 100,000 people [53].

A myriad of studies on the effects of MSCs in treating MSA has shown positive results. Intra-arterial delivery of BM-MSCs to patients with cerebral type MSA. Different doses of MSCs were administered to the patients (low dose: 3 × 10^5^, medium dose: 6 × 10^5^, and high dose: 9 × 10^5^ cells/kg body weight of the patient). The results show that the medium and high doses showed a slower increase in the Unified MSA Rating Scale score (lower score = improvement), showing that MSCs can protect the patients from neurodegeneration [54]. The long-term effect of UCB-MSCs was studied in MSA patients who received the cells through lateral atlanto-occipital space puncture. The Unified MSA rating scale was used to assess the patients for 3–5 years. UCB-MSCs seemed to ameliorate MSA with no adverse effects. However, the greatest effect of MSCs on MSA was observed 3–6 months after the first dose [55]. Another important study documented improvement in behavioral disorders and prevention of neurodegeneration in patients on a toxin-induced multiple system atrophy model which was attributed to the likely reduction of polyamine-induced and cholesterol-induced damage mediated by BM-MSCs trophic and reparative functions [127]. Clinical research involving intrathecal administration of 10 and 200 million autologous Ad-MSCs to 24 patients with multiple system atrophy resulted in lower rates of disease progression, as assessed by UMSARS score without any side effects [128]. Of particular significance in cellular therapy are studies that examine dosage safety and optimize administration route. Delivery of higher doses of BM-MSCs (2 × 10^9^) through the internal carotid artery in an animal model of MSA was found to be lethal to the animal. Animals that received a comparatively lower dose, i.e., 8 × 10^7^ MSCs, had a greater survival rate of nigrostriatal neurons without any side effects [56]. Research investigations to determine optimal dose and delivery must be carefully considered to ensure safety and efficacy of MSC therapies.

#### Amyotrophic lateral sclerosis

ALS is a disease that selectively affects motor neurons, weakening muscles and impacting physical functions, eventually leading to progressive paralysis of most voluntary muscles. It affects as many as 30,000 people in the United States, with 5,000 new cases diagnosed each year; being a degenerative disorder, ALS is most common in adults 60 years and older [57, 58].

Studies on the effects of MSCs in ALS demonstrated safety and efficacy in the treatment strategies tested. Two injections in a month interval of an average of 0.42 × 10^6^ WJ-MSCs to the cervical, lumbar, and thoracic regions of 43 ALS patients did not show any adverse effects confirming the safety of MSC administration [59]. Phase I clinical trials investigating effects of single-dose intramuscular or intrathecal administration of autologous BM-MSCs or Ad-MSCs secreting neurotrophic factors, followed by both intramuscular and intrathecal injection in Phase II of the study showed no negative secondary effects. Moreover, the rate of the ALS Functional Rating Scale-Revived score and the forced vital capacity lowered in patients administered with MSCs intrathecally (accounting for the combination of intrathecally and intramuscularly) [60]. In another study, survival time increased in 67 subjects administered with three intrathecal injections of 30 × 10^6^ WJ-MSCs [129]. BM-MSCs in combination with Riluzole seem to reduce ALS disease progression of subjects after 4 and 6 months compared to the control group (Riluzole alone). The ALS Functional Rating Scale was reduced in the treatment group. The MSC-treated group also showed an increase in anti-inflammatory and a decrease in pro-inflammatory cytokines. There was no difference in treatment-related adverse effects between the 2 groups [61]. Another study administered BM-MSCs induced to secrete high levels of neurotrophic factors to patients with ALS. Results showed an improvement in neurodegeneration, neuroinflammation, and neurotrophic factors as shown in the cerebrospinal biomarker analysis [62], demonstrating their potential to be applied as suitable therapeutic candidates for treating ALS.

#### Spinal cord injury

SCI is the result of damage to the spinal cord, which results in a loss of function, feeling, and mobility. The spinal cord does not have to be severed for a loss of function. About 17,900 new cases of SCI are diagnosed every year, with 78% being male patients [63]. Currently, there is no cure for SCI; however, new advances in research continue to develop in research and translational laboratories.

MSC transplantation continues to be explored as noninvasive treatment options to regenerate injured spinal cords. Clinical trials data support their usefulness for treatment; specifically, intravenous injection of UC-MSCs (1 × 10^6^/kg) each month for four months in early-stage SCI patients improved neurological dysfunction and was found to be safe and effective in restoring quality of life as observed in 1-, 3-, 6-, and 12-months post-treatment follow-up. Motor and sensory functions notably improved, along with improvements in the overall mobility of the patients [64]. In a related study, administration of NeuroRegen scaffold with UC-MSCs, via a small incision in eight SCI patients under general anesthesia, improved the motor and sensory functions of the patients without any side effects, as seen in a 1-month follow-up [65]. Likewise, intrathecal injection of 9 × 10^7^ Ad-MSCs through lumbar tapping in 14 patients showed improved motor and sensory skills 8 months post-treatment. However, further research is underway to verify the correct dosage needed [130]. Studies demonstrating the safety and feasibility of MSC therapy indicate that intrathecal administration of autologous BM-MSCs (2–3 total injections) in six patients did not produce any negative effects [131]. In a similar trial, researchers labeled UC-MSCs with aggregation induced emission-Tat nanoparticles to track how effective MSC treatment was for SCI. A rat model was used to track the MSC therapy effectiveness. The results showed no adverse effects as well as SCI recovery showed by histopathological and behavioral rehabilitation results. Moreover, the cells were able to conserve the cell viability in microenvironments with highly reactive oxygen species and produce nerve growth factors [132].

MSC utility and its therapeutic merits for neural repair and regeneration are supported by several preclinical and clinical trials, together offering more effective treatment approaches for neurodegenerative disorders.

### Cardiovascular applications

Cardiovascular diseases are a major cause of death in the United States with 1 in every 4 deaths caused by heart disease [66]. Although there are numerous efforts in scientific community to find effective treatments, researchers are focusing on MSCs due to their coveted regenerative properties and ease of isolation and expansion. Currently, MSC application in-vitro and in-vivo cardiovascular disease treatments have shown ameliorating results indicating promising therapeutic potential. In this section, an overview of MSCs used in hypertension, myocardial infarction, stroke, heart failure, and chronic ischemic cardiomyopathy is presented.

#### Hypertension

Pulmonary arterial hypertension can lead to elevated arterial pressure and increased pulmonary vascular resistance. The World Health Organization estimates that about 1.28 billion adults within the age range 30–79 have hypertension worldwide [67]. Moreover, there is no cure for the disease to date.

Of the several treatment options being carefully considered for hypertension in recent years, MSCs have emerged as suitable therapies based on preclinical evidence in attenuating hemodynamic and histological progression of pulmonary arterial hypertension [68]. Human MSC-derived extracellular vesicles restored right ventricular systolic pressure to baseline levels; and reversed right ventricular hypertrophy and peripheral pulmonary artery muscularization on a rat Sugen/hypoxia model with pulmonary hypertension [69]. Injection of extracellular vesicles from iPSC-derived MSCs through the mice tail vein reduced arterial stiffness and hypertension; and promoted the expression of AMPKα, eNOS, and SIRT1 [70]. Similarly, BM-MSC transplantation on a chronic hypoxia-induced pulmonary hypertension rat model improved collagen deposition, decreased muscularization, thickening, and pulmonary arterial pressure of the disease carrier. MSCs also attenuated EndMT and factor-2α (hypoxia-induced factors) [133]. Additionally, treatment with skin-derived MSCs decreased vascular damage and systolic blood pressure; reduced Th17 cells in peripheral blood; and lowered the levels of protein, and IL-17 mRNA on the aorta and serum in an AngII-induced hypertensive mouse model. MSCs also switched macrophages to an anti-inflammatory profile (M1 to M2) and increased the rates of migration and proliferation of MSCs [71]. In another study, human UC-MSC-derived exosomes showed promising results both when injected into the monocrotaline-induced pulmonary hypertension rat model and hypoxia-induced cell model. MSC exosomes lessened hypertrophy in the right ventricle and caused pulmonary vascular remodeling. It was further noted that pulmonary arterial smooth muscle cell proliferation and pulmonary arterial endothelial cell apoptosis were inhibited, while increases in Wnt5a expression and suppression of endothelial to mesenchymal transition (EndMT) factor were observed [73].

#### Myocardial infarction

Myocardial infarction is the primary cause of disability and death in patients affected by this disorder which is normally treated as a medical emergency. It is characterized by cardiomyocyte death induced by cardiac ischemia [72]. Since no effective treatments are available, research is underway to explore regenerative approaches for tissue repair and regeneration.

Several clinical trials have tested MSCs for treating myocardial infarction, and the results have thus far been promising. Exosomes from Ad-MSCs suppressed cardiac dysfunction, fibrosis, and apoptosis on myocardial infarction-induced cardiac damage using H9c2cells, HAPI cells, and cardiac fibroblasts. Also, a decrease in the inflammatory response, an increase in macrophage M2 polarization, and activation of sphingosine 1 phosphate signaling, S1P/SK1/S1PR1, was observed [74]. Furthermore, intramyocardial injection of synthetic MSCs into a myocardial infarction mouse model showed improvement in angiogenesis and remodeling of the left ventricle. Synthetic MSCs are produced by inserting factors secreted by BM-MSCs into poly(lactic-co-glycolic acid) microparticles covered with the MSC’s membrane structure [72]. A related study documented that EVs secreted from SDF1 overexpressing MSCs promoted the microvascular restoration of endothelial cells and inhibited apoptosis of myocardial cells in a myocardial infarction mouse model. The secretion of these exosomes, as seen by coculture experiments, was interrupted by the neural sphingomyelinase inhibitor GW4969, whereas it was promoted by the SDF1 plasmid [134]. Similarly, anti-miR-155-5p BM-MSCs improved angiogenesis and cell survival in a myocardial infarction mouse model. It was noted that anti-miR-155-5p MSCs resulted in improvement in cardiac function in comparison to the MSCs alone [75]. Exosomes derived from human UC-MSCs that overexpressed TIMP2 enhanced cardiac function and promoted angiogenesis at the myocardial infarction site on a myocardial infarction rat model. In addition, MSCs alleviated oxidative stress produced by myocardial infarction and ECM restoration, with significant involvement of the Akt/Sfrp2 pathway in mediating cellular responses to MSC intervention [76].

#### Stroke

Stroke is the resulting damage to the brain due to interruption of blood supply. It can be either ischemic (caused by the lack of blood flow) or hemorrhagic (caused by bleeding). Finding effective treatments for stroke is a clinical need as it continues to remain as one of the leading causes of death worldwide [77].

Multiple clinical trials involving MSCs and MSC-derived extracellular vesicles have suggested that MSC-based regenerative therapy could lead to effective stroke therapy [78]. Intravenous injection of exosomes from BM-MSCs enhanced recovery of neurological function, improved neurogenesis and angiogenesis, and reduced IL-1β expression in a mouse model of ischemic stroke [79]. Similarly, human UC-MSC-derived exosomes reduced inflammation in vitro in microglia-mediated neuroinflammation after experiencing ischemic stroke. Additionally, they decreased behavioral defects, lowered infarct volume, and enhanced activation of microglia. These neuroprotective effects of MSC-derived exosomes, however, were partially undone by miR-146a-5p [135]. In a related clinical work, administering MSCs from various sources as adjuvant therapy to the standard treatment for patients with severe infarction of the middle cerebral artery greatly improved motor functions in the lower extremities without any side-effects compared to groups that only received the standard treatment [80]. MicroRNA-184 and microRNA-210 present in rat BM-MSC-EVs were found to be the key factors in promoting angiogenesis and neurogenesis in a stroke model. Also, MSC-EVs were more efficient in the improvement of behavior than whole parental MSCs [81]. Exosomes derived from BM-MSCs improved neurological function, reduced weight loss after stroke, and enhanced neuroprotective effects in type 2 diabetic stroke rat model compared to control. These exosomes caused remodeling of white matter and anti-inflammatory responses. Additionally, it was found that the treatment decreased miR-9 expression, which increased the ABCA1 pathway [82].

#### Heart failure

Congestive heart failure is a chronic condition characterized by the inadequate pumping of blood. Though this disease lacks effective treatments, some symptomatic treatments that can increase survival are currently available [83].

Many studies have been conducted to elucidate the applications of MSCs in the treatment of heart failure, with hopes of developing effective treatments. Administration of allogeneic UC-MSCs into patients with congestive heart failure increased the expression of hepatocyte growth factor (associated with immunomodulation, myogenesis, and cell migration), and enhanced the ejection in the left ventricle [84]. Similarly, MSCs overexpressing adrenomedullin (ADM) enhanced heart function and increased cell survival in a rat model of heart failure. This was accompanied by decreased expression of matrix metalloproteinase-2 and the fibrotic area percentage; and significantly influenced ADM and hepatocytic growth factors as compared to the rat model treated with the normal MSCs [136]. We also note that superparamagnetic iron oxide nanoparticles-labeled human amniotic MSCs increased cell homing and enhanced myocardial hypertrophy and heart function in the presence of a magnetic field on a heart failure rat model. In addition, a reduction in fibrosis was also seen in comparison to the group tested in the absence of the magnetic field [85]. Interestingly, preclinical work showed methods to increase AD-MSC therapeutic efficiency in a mouse model of pressure overload heart failure. The study showed that enhancing the MSCs exosomes through treatment of adiponectin improved cardiac function and reduced inflammation and fibrosis in the mouse model [86]. Supporting studies on intravenous BM-MSCs injection into a rat heart failure model demonstrate the efficacy of MSCs in reducing myocardial infarction size and interstitial fibrosis and enhancing heart rate variability and baroreflex sensitivity [87].

#### Chronic ischemic cardiomyopathy

Chronic Ischemic Cardiomyopathy (CIC) is characterized by a significant reduction in the heart’s ability to pump blood either due to the main pumping chamber of the heart being enlarged or dilated due to a lack of blood supply to the heart. It is one of the most common cardiovascular disorders [88].

Clinical trials with MSCs have yielded positive outcomes suggesting that cellular therapies involving MSCs may prove useful to treat CIC. A study (*n* = 50) demonstrated that injection of UC-MSCs lowered the percentage of infarct size change [89]. Transplantation of either BM-MSCs or UC-MSCs with a Coronary Artery Bypass Grafting Surgery (CABG) showed a decline in NT-proBNP in 1, 3, 6, and 12 months follow-up as compared to the patients receiving CABG intervention alone. It was noted that the UC-MSC group had an increase in left ventricular ejection fraction (LVEF) in comparison to the control group [137]. Similarly, patients receiving BM-MSC infusion with revascularization showed improvement in left ventricle function in comparison to the group who only received the revascularization, as well as patients who received BM-MSC infusion via intracoronary administration. Together, these results demonstrate that MSC infusion significantly increases and improves cardiac function in CIC [138]. In a similar study, intramyocardial injection of either UC-MSCs or BM-MSCs along with coronary artery bypass grafting (CABG) surgery improved left ventricle function in patients as seen in 1, 3, 6, and 12 months follow-up. However, further tests and parameters must be considered before effectiveness of such treatment can be confirmed [139]. Another study documented the effects of transendocardial and intramyocardial injection of BM-MSCs in the left ventricle scar of the patients in significantly improving the regional function of the left ventricle in 3- and 6- months follow-up [140].

Overall, MSC treatment potential for cardiovascular diseases can be attributed to their immunoregulatory ability, anti-fibrotic and anti-scarring effects, angiogenic, and neovascularization functions.

### Immune-related applications

The immunoregulatory properties of MSCs can influence both innate and adaptive immune responses. MSCs inhibit T-lymphocyte proliferation that is induced by mitogenic and by allogeneic peripheral blood lymphocyte (PBLs) and dendritic cells (DCs) [141]. This T cell inhibition is dose-dependent with the peak significant reduction in T-lymphocyte proliferation. MSCs have also been seen to influence the formation of regulatory T cells that help with the inhibition of allogenic lymphocyte proliferation. Even though the exact mechanism for MSC immunosuppressive effects remains to be fully explored, we note from established literature that soluble factors such as prostaglandin E2 (PGE2)], indoleamine2,3-dioxygenase (IDO), hepatocyte growth factor (HGF), and transforming growth factor (TGF)-b1 play a major role in the immunosuppressive effects of MSCs. MSCs are known to involve in the generation and development of DCs, which are antigen-presenting cells (APC) in the immune system. Furthermore, MSCs in coculture with DCs were found to reduce the expression of CCR7 by the DCs and inhibit the differentiation of monocytes to DCs [90]. MSCs are also able to promote M1-to-M2 phenotype transformation of macrophages. Macrophages are specialized immune cells involved in the detection, phagocytosis, and removal of pathogenic agents in the innate immune system. Macrophage differentiation from monocytes occurs in the tissue in response to microenvironmental signals resulting in acquisition of specific functional phenotypes [91]. MSCs mediate the regulation of macrophages, which is crucial for limited inflammation response and damaged tissue healing. BM-MSCs have been known to regulate the host’s inflammatory response to sepsis and significantly improve the host’s survival [90]. The roles of MSCs in regulating various immune cell types pose them as suitable therapeutic candidates for treatment of immune-related diseases. Common immune related disorders that are targeted for MSCs therapy include autoimmune type 1 diabetes mellitus, rheumatoid arthritis, systemic lupus erythematosus, and graft vs. host disease, which will be discussed below.

#### Type 1 diabetes mellitus

The pancreatic islet houses a group of cells, such as beta (β) cells, to synthesize insulin [137]. The progressive autoimmune attack on pancreatic β-cells results in the loss of insulin secretion and production, ultimately affecting the blood sugar levels and metabolic functions resulting in a condition termed type 1 diabetes mellitus (T1D) [92]. As of 2020, about 34.2 million people of different ages were diagnosed with T1D [93].

Although insulin injections are effective in glycemic control, they fail to address the other side effects of T1D, such as nephropathy, high blood pressure, and foot diseases [138]. Thus, a permanent cure to T1D must address the autoimmune response, followed by an emphasis on islet regeneration and replacement. Therefore, MSC transplantation are thought to be possible treatments for this disorder owing to their engraftment with differentiation and trophic effects. A trial showed that intrapancreatic transplantation of BM-MSCs decreased blood glucose and increased C-peptide and insulin in a rat model. This suggests that MSCs can improve damaged pancreases function [94]. In another trial, the mechanism by which MSCs aid in T1D recovery has been studied in a diseased mice model. human UC-MSCs were used in this study. The results showed that TGFBI was crucial for MSC immunosuppressive capacity an suppression of activated T-cell proliferation [142]. BM-MSCs were shown to be a better treatment than platelet-rich plasma injection in T1D rat model. The results showed that the group treated with BM-MSCs had an increase in the diameter of Langerhans islets and the amount of zymogen granules compared to the untreated group and the group treated with platelet-rich plasma [95]. A study tested the immunomodulatory capacity and beta cell protection of Ad-MSCs in a diabetic mice model. This study showed that Ad-MSCs can maintain the secretion of insulin and the viability of pancreatic islets when reactive splentocytes are present. Moreover, Ad-MSCs decreased splentocyte proliferative response [96]. A similar studied the use of human UC-MSCs that were previously modified to express exenatide in a T1D mice model. The results showed that UC-MSCs aided in damaged islet tissue repair. These cells also decreased the renal tissue lesions as well as the blood glucose levels. Moreover, there was less pro-inflammatory and more anti-inflammatory intestinal bacteria [143].

#### Rheumatoid arthritis

Rheumatoid arthritis (RA) is an autoimmune and inflammatory disorder where the immune system attacks the healthy cells, causing swelling and inflammation of joints. RA can also spread to other areas of the body and is mostly diagnosed in females [97]. The causes of RA are yet to be fully understood.

MSC administration, in RA patients, has shown positive outcomes in recent clinical trials. Administration of UC-MSCs (4 × 10^7^cells total dose) to 64 patients diagnosed with RA resulted in a significant decrease in blood globulin and blood platelet levels during 1-year and 3-year post-treatment follow-up. In addition, there was a significant decrease in the erythrocyte sedimentation rate (ESR), C-reactive protein levels, and rheumatoid factor (RF). This decrease in serological and immunological markers in patients with RA and high immune system activity highlights MSC therapeutic potential and immunomodulation. Long-term patient relief and joint function (measured by the DAS28 score) also showed a significant decrease in these levels post MSC treatment at 1-year and 3-years [98]. Similarly, a clinical trial investigated 105 patients showing little to no responses to traditional RA drugs, such as disease-modifying antirheumatic drugs (DMARDs) and non-steroidal anti-inflammatory drugs (NSAIDs), to study the effects of MSC transplantation. Patients were divided into control group and MSC transplantation group (MSCT). The MSCT group was intravenously infused with UC-MSCs (1 × 10^6^ cells/kg in a 50 ml saline solution) and followed up at 1, 2, 3, 4, 12, 24, and 48 weeks. Of the 52 patients in the MSCT group, 28 patients were observed to have a response to the MSC treatment after 12 weeks and a significant decrease in the Disease Activity Score-28 (DAS28). Also, patients in the control group and those non-responsive to the MSC treatment did not show any significant changes in DAS28, together indicating that the MSC treatment improved overall clinical symptoms [99]. RA patients typically exhibit a low percentage of CD4^+^CD25^+^Foxp3^+^ Tregs and a high percentage of CD4^+^IL-17A^+^ Th17 cells. Thus, a low ratio between Tregs to Th17 cells indicates an imbalance in the immune homeostasis [144]. This study showed a significantly high Treg to Th17 ratio in the response group, as compared to the control and non-response groups, showcasing UC-MSC immunomodulatory effects on deregulating RA development. Additionally, there was a significant decrease in IL-6 and a significant increase in IL-10 levels at the 4-weeks checkup. Typically, RA patients are observed to have low IL-10 and high IL-6 levels [99]. A clinical trial in phase 1a tested MSC as a treatment for RA in humans. Human UCB-MSCs were infused into patients with RA. These patents received a single infusion of MSCs. Serum cytokines were measured to determine the effectiveness of the treatment after 24 h. The results showed a decrease in IL-8, IL-6, IL-1β, and TNF-α in the group that received the higher cell dose (1*10^8 cells). Moreover, No adverse or safety threats were observed short-term [100]. A study aimed to investigate the molecular mechanisms by which BM-MSCs ameliorate RA symptoms. This study showed that the cells effectively decreased microRNA-584e levels through reduction of NF-κB activity. Mice models were used for this study [101]. Finally, implantation of BM-MSCs (40 million BM-MSCs per joint) in RA patients with a knee injury showed greater improvements in their knee injury than the placebo group that received normal saline instead of MSCs [145]. Although BM-MSC transplantation treatment may be a beneficial for RA patients, research with more patients must be done.

#### Systemic lupus erythematosus

Systemic Lupus Erythematosus (SLE) is an autoimmune disorder and the most common form of lupus. According to the Centers for Disease Control and Prevention (CDC), SLE is a disorder in which the immune system attacks healthy tissues, causing widespread inflammation and tissue damage in organs. It can affect the lungs, blood vessels, joints, brain, kidneys, and skin. More women are diagnosed with SLE compared to men: the ratio is at least 4 women to every 1 man [102].

While there is no cure for SLE, MSC transplantation, in recent clinical trials, shows promising results as a novel cell therapy. Intravenous injection of allogeneic UC-MSCs in 39 patients with active SLE twice in a 1-week interval showed a significant decrease in the SLE disease activity index (SLEDAI) score in 1, 3, 6, 9, and 12 months follow-up. Additionally, improvement in the levels of serum albumin was seen at the 1-month follow-up, which was maintained up to 12 months, after which it declined [103]. This indicates a possibility of disease relapse thus, repetitive UC-MSC infusions after 6-months should be considered. In another study, BM-MSCs of passages between 3 and 5were intravenously injected (1 × 10^6^ cells BM-MSCs/kg body weight) in 15 patients (14 women and 1 man) of an average age of 28 years and a SLEDAI score greater or equal to 8. Then the measurement of regulatory T cells (Tregs), urinary protein excretion, glomerular filtration rate (GFR) assessments, and serological testing was done after 1-week, 1, 3, 6, 12, and 18 months followed by once every half a year post-transplantation. Overall improvement in SLE was observed: SLEDAI scores significantly improved on the 12-month follow-up, serological tests (ANA and anti-dsDNA antibodies) showed substantial improvement, anti-dsDNA antibody significantly decreased from the baseline at 1-month and 3-month follow-up, proteinuria significantly decreased at the 1-week,1-month, 3-months, 6-months, and 12-month examination, and GFR improved significantly in two patients with a low baseline GFR. Likewise, there was a significant increase in CD4^+^Foxp3^+^ Treg cell percentage at the 1-week, 3-month, and 6-month examinations [104]. This increase in CD4^+^Foxp3^+^ Treg cell percentage suggests a possible Treg expansion caused by the BM-MSCs, which helped maintain immune self-tolerance. CD4^+^Foxp3^+^ cells are a subset of the CD4 T-cells that are involved in modulating immune-homeostasis and immune cell activation. Decreased regulatory T cells (Tregs) can be related to the progression of human autoimmune diseases [146] Measurements of the CD4^+^Foxp3^+^ cell percentage in peripheral blood mononuclear blood cells can be assessed to examine the role of MSCs in Treg regulation. Transplantation of UC-MSC followed by intravenous injection of a dose of prednisone (5–10 mg) every two weeks for the first month showed an increase in Treg cells and a balance between Th1 and Th2 cytokines, ultimately causing the SLE activity in the body to decrease, without any signs of possible relapse in 1 and 3 months follow-up [105]. Another study highlighted the possibility that a baseline serum could correlate with more efficient MSCs. In this study, 56 active SLE patients and 40 healthy patients were enrolled. Of these 96 patients, 26 were administered UC-MSCs intravenously. After 1 year following the transplant, 17 patients showed a clinical response to the UC-MSCs, whereas 9 patients did not. The baseline serum cytokines were analyzed following the transplant, and the results were correlated to the clinical responses. This study showed that increased levels of IFN-γ and decreased levels of IL-6 can be used as a serological marker for efficiency of MSC treatment of SLE since these serological findings correlate with clinical responses of patients administered with UC-MSCs intravenously. However, further research must be done to validate this connection [106]. Finally, in vitro study with 24 SLE patients and 28 healthy patients aimed to see the effects of UC-MSC on inflammatory factors of SLE patients based on the T lymphocyte. T lymphocytes extracted and sorted using Miltenyi magnetic beads, along with IL-2 and CD3CD28 T-cell activators, were co-cultured with UC-MSCs. Results showed that UC-MSCs might be able to upregulate miR-181a, a gene expression for T cells, while down-regulating inflammatory genes, thus promising to be beneficial for SLE treatments [107].

#### Graft vs. host disease

Graft vs. host disease (GVHD) is an autoimmune condition that occurs after allogeneic transplantation and is life-threatening. GVHD occurs when the transplant regards the body as foreign and starts to attack it causing complications for the patient. It is less likely to occur if the donor and the patient are close tissue and cell matches [108].

There is no effective treatment for GVHD; however, clinical trials with MSCs have shown promising outcomes. A study administered BM-MSCs to 9 patients experiencing GVHD after allogeneic transplantation of bone marrow. Five patients received a single dose of MSCs while the other received 2–6 doses. The effects of this therapy were measured at 14 days and 28 days. Remission of the GVHD (partial or complete) was obtained in 56% of patients after the first dose and in 44% after all the doses administered. There were no significant side effects of the MSC therapy long-term (4–8 year follow-up) [109]. In addition, the number of Th1 cells in acute GVHD patients decreased after the administration of BM-MSC therapy [147]. Similarly, Human Leukocyte antigen-haploidentical (HLA-haploidentical) hematopoietic stem cell transplantation in chronic GVHD (cGVHD) completely cured cGVHD until 100 days after the transplant. Here, 124 patients from different transplantation centers participated in the study, with 62 patients randomly assigned to the saline infusion (control) and other 62 patients to HLA-haploidentical) hematopoietic stem cell infusion. An HLA-haploidentical) hematopoietic stem cell dosage of 3 × 10^7^cells/100 ml per month was administered for 4-months. In the treatment group, cGVHD developed in 17 patients, while 30 patients developed cGVHD in the control group. In addition, 7-patients developed severe lung cGVHD in the control group, while none of the patients developed lung cGVHD in the treatment group. Furthermore, flow cytometry analysis showed that CD4^+^CD25 + CD127 − regulatory T (Treg) cells were higher in treatment groups compared to the control group. This increase in Treg cells might have suppressed the occurrence of cGVHD in the treatment group. Additionally, there was a decrease in Natural-Killer (NK) cells after HLA-haploidentical) hematopoietic stem cell injections, which indicates a direct relationship between NK cell count and the occurrence of cGVHD [148]. Likewise, 46 patients with steroid-refractory acute GvHD (aGvHD) grade III/IV were treated with BM-MSC infusions with a median cumulative dose of 6.81 × 10^6^ cells/kg at 7-day intervals. Of the 46 patients, 23 showed a response to the MSC treatment. These patients had a more significant overall survival (OS) time than the nonresponses. Furthermore, 7 patients were alive at 48.07 months after MSC infusion. The OS rates for the responders at the 1- and 2-year mark were 19.56% and 17.4%, respectively. Compared to the nonresponses OS rates, it was approximately 0% at the 1-and 2-year mark. No patients exhibited a severe side effect of the MSC infusion, suggesting BM-MSC infusion is safe for GvHD III/IV patients [149]. In another trial, cGVHD patients received repeated BM-MSCs infusions which resulted in 6 out of 11 patients responding to therapy according to the National Institutes of Health criteria. MSC treatment showed an increase in B cells and Tregs 7 days after each infusion. This study affirmed that MSC treatment is safe with durable responses for GVHD [150].

## Conclusion

Due to their multilineage differentiation, proliferation capacity, secretive factors, and relative ease of isolation from different body parts, MSCs can be applied in numerous conditions. Moreover, there are active efforts to optimize MSC administration and disease treatment techniques. Many clinical trials have used different MSC doses, compared the effectiveness of MSCs from different sources, used MSC’s exosomes instead of the actual cells, and combined MSCs with other substances/cells. Though this review paper focused on the diseases compromising the musculoskeletal, nervous, cardiovascular, and immune systems, clinical trials with MSCs are not limited to these four systems, and they are being tested in many more areas. Further research is, therefore, required in regenerative medicine for MSCs to effectively treat diseases/disorders.

## Data Availability

Please contact corresponding author for data requests.
